# Use of polydimethylsiloxane topomimetic artificial blueberry, strawberry, and raspberry surfaces for the study of foodborne virus recovery and inactivation in berries

**DOI:** 10.3389/fmicb.2026.1807746

**Published:** 2026-04-30

**Authors:** Ashlyn Lightbown, Erin DiCaprio

**Affiliations:** Department of Food Science and Technology, University of California, Davis, Davis, CA, United States

**Keywords:** berries, peracetic acid, polydimethylsiloxane, replicasts, sodium hypochlorite, surface topography, Tulane virus

## Abstract

Foodborne viruses have caused large-scale outbreaks in fresh and frozen berries, such as strawberries, blueberries, and raspberries. Studies have previously evaluated the use of non-thermal technologies (including chemical sanitizers) to inactivate foodborne viruses on the surface of soft berries with varying results. In this study, we investigate a novel model system for utility in characterizing virus attachment and removal, as well as sanitizer inactivation on the surface of berries. A casting method using polydimethylsiloxane (PDMS) was used to create topomimetic artificial surfaces (“replicasts”) of strawberries, blueberries, and raspberries to elucidate the impact of berry surface structures (topography) on virus adhesion and inactivation. A human norovirus surrogate, Tulane virus (TV), was inoculated (2 × 10^5^ log_10_ PFU) on the surface of fresh and replicast berries, and viral recovery levels were compared. Overall, the type of berry impacted viral recovery, with 5.11 log_10_ PFU recovered from blueberries, 5.04 log_10_ PFU recovered from strawberries, and 5.66 log_10_ PFU recovered from raspberries. Surface inoculation of blueberry, strawberry, and raspberry replicasts with TV led to comparable recovery levels of virus as observed in fresh berries, with 4.80, 4.93, and 5.17 log_10_ PFU recovered, respectively, with a two-step recovery procedure. Sodium hypochlorite (200 ppm) led to less than a one log_10_ PFU reduction in viral titer in all three types of fresh berries. However, the same sanitizer treatment and two-step recovery procedure led to a 2.45 log_10_ PFU reduction in blueberry replicasts, 2.24 log_10_ PFU reduction in strawberry replicasts, and 1.59 log_10_ PFU reduction in raspberry replicasts. Sodium hypochlorite (50 ppm) and peracetic acid (20 or 80 ppm) had limited efficacy in inactivating viruses inoculated to fresh berry or berry replicast surfaces. However, viral reductions were greater in replicasts compared to fresh berries when observed. These data indicate that while berry replicasts may have utility in modeling surface adhesion of viruses to berries and in viral recovery studies, they are not suitable for studies of viral inactivation by chemical sanitizers.

## 1 Introduction

As the leading causative agent of foodborne outbreaks, human norovirus (HuNoV) is estimated to cause 58% of foodborne illnesses reported in the United States each year (U.S. Centers for Disease Control and Prevention, 2024). Foodborne viruses cannot replicate in the agri-food supply chain yet pose a significant food safety risk. All foodborne viruses are non-enveloped, improving their resistance to various environmental stressors and chemical sanitizers as compared to enveloped viruses or vegetative bacterial cells (Baert et al., 2009a; Koopmans and Duizer, 2004). Therefore, foodborne viruses may persist for long periods of time in food or the production environment after contamination (Jaykus et al., 2012). Previous studies have demonstrated that water-washed produce (with and without a chemical sanitizer supplement) results in limited inactivation or removal of foodborne viruses from produce surfaces (Baert et al., 2009b; Bosch et al., 2018).

The joint 2024 FAO/WHO Expert Meeting on the microbiological risk assessment of viruses in foods released pairings of high-risk foods for virus contamination based on frequency of illness and clinical severity of disease, ranking frozen berries as the second-highest risk commodity for HuNoV-associated outbreaks (FAO and WHO, 2024). The determination of high-risk foods for virus contamination was based on the “highest global public health burden” (FAO and WHO, 2024), and the most recent outbreaks in the United States caused by foodborne viruses have been associated with imported fresh and frozen berries (FDA, 2025). The causative agents of berry outbreaks between 1983 and 2018 have shifted from parasitic to predominantly viral, namely HuNoV and hepatitis A virus (HAV). In this time period, there were 68 reported outbreaks globally associated with viral contamination of berries, leading to over 18,000 illnesses (Bozkurt et al., 2021). Of these 68 berry outbreaks, HuNoV was implicated in 46. Frozen berries were responsible for 50 of the 68 outbreaks, with HuNoV causing 36 and HAV causing 14. Frozen raspberries were responsible for more than 80% of HuNoV outbreaks, and frozen berry mix was responsible for the majority of HAV outbreaks (44%) (Bozkurt et al., 2021).

Factors contributing to berry contamination were identified in 8 of 46 (17%) HuNoV outbreaks, with food handlers implicated in 75%, contaminated water in 12.5%, and human harvesters in 12.5% (Bozkurt et al., 2021). Berries are often consumed fresh with no or minimal processing after they are picked and packed manually, remaining unwashed to maintain quality. Food handlers and contaminated food contact surfaces pose a risk of berry contamination with viral pathogens, as postharvest contamination of berries can occur from contaminated surfaces, water, or direct contact with infected individuals (FDA, 2025). Washing, use of chemical sanitizers, freezing, and frozen storage have limited efficacy in inactivating enteric viruses on berries (Butot et al., 2008; Maks et al., 2019).

Variability between plants is an intrinsic limitation to using fresh plant tissue in experiments. As a tool to mitigate variation, previous study has shown that topomimetic artificial leaf surfaces, that is, replicated casts (“replicasts”) of real leaves made from polydimethylsiloxane (PDMS), can be used in place of fresh plant tissue to characterize the interaction between foodborne viruses and produce surfaces (Sun et al., 2019). Microscopic comparison of fresh leaves and their PDMS replicasts revealed high-fidelity replication of various leaf structures, including veins, epidermal pavement cells, trichomes, and stomata (Doan et al., 2020b). PDMS replicasts are also compatible with a wide range of microscopy techniques and various sterilization and decontamination practices that aid in experimental use and re-use of replicasts (Doan et al., 2020b). Finally, surface chemical properties are standardized with PDMS replicasts, thus allowing for isolation of surface topography from surface hydrophobicity effects (Sun et al., 2019).

To better understand the factors contributing to the persistence of foodborne viruses on fresh produce surfaces, various mechanisms for characterizing the interactions between produce and viruses have been proposed and studied. These include non-specific binding of the viral particle to produce surfaces through ionic interactions, specific receptor-mediated attachment of the virus to produce surface moieties, or purely physical interactions relative to the size of the viral particle and the surface complexity of the produce. Previous studies have demonstrated that the isoelectric point and pH of the surrounding medium will cause the viral capsid to have an overall net positive or negative charge, and modulating the viral capsid electrostatic characteristics can alter absorption and desorption to produce surfaces (Tian et al., 2011; Vega et al., 2008; Vega et al., 2005). The cellular receptor recognized for HuNoV attachment is the histo-blood group antigens (HBGAs). Previously, HuNoV genogroup II genotype 4 (GII.4) virus-like particles (VLPs) were found to attach to H-type HBGA-like carbohydrate components of romaine lettuce cell walls, and this attachment was mediated by the HuNoV HBGA binding domain (Gao et al., 2016; Esseili et al., 2019). In contrast to leafy greens, it has been proposed that ionic interactions are primarily responsible for the binding of HuNoV to raspberries. Distribution of HuNoV VLPs was found to be at random without localization or strong attachment to any specific area of the raspberry surface (Tian et al., 2011). However, the role of microscale complexity of berry surfaces on adhesion and dispersion of viruses has not been directly compared.

Previous studies utilizing PDMS replicasts for microscale surface topography evaluation have been conducted with leafy green templates (Doan et al., 2020a,b; Yi et al., 2022; Lightbown and DiCaprio, 2025). By using PDMS replicasts of berries, the impact of berry surface microscale topography can be studied without the influence of variables like native microbiota, plant cuticle characteristics, and other plant surface moieties. The aim of this study was to utilize PDMS replicasts of blueberry, strawberry, and raspberry surfaces to understand the impact that physical structures on berry surfaces have on virus recovery and inactivation via washing with and without chemical sanitizers.

## 2 Materials and methods

### 2.1 Fabrication of PDMS replicasts

Conventionally grown blueberries, strawberries, and raspberries in clear, vented clamshell containers were purchased from the local supermarket in Davis, CA, in the fall of 2024. Three to six berries were randomly selected for each species and then allowed to acclimate to room temperature. PDMS replicasts of blueberry, strawberry, and raspberry surfaces were fabricated in a previously described two-step molding process (Sun et al., 2019; Lightbown and DiCaprio, 2025) with some modification for berries as compared to the casting process for leafy greens. In brief, the SYLGARD™ 184 Silicone Elastomer Kit (Dow Chemical Company, Midland, MI, U.S.) was used to create negative molds of fresh berries. After curing for 48–72 h at room temperature, the negative molds were separated from the fresh plant tissue and cleaned with a 1:100 solution of Triton™ X-100 (Sigma-Aldrich, St. Louis, MO, U.S.). These negative molds were treated with UV light for 60 min before submersion in a 500:1 solution of toluene to octadecyltrichlorosilane (Sigma-Aldrich, St. Louis, MO, U.S.) for 5 min. Negative molds were rinsed with absolute ethanol for another 2 min before drying completely. PDMS was poured onto the prepared negative molds and allowed to set at room temperature for 48 h. The positive cast was then separated from the negative mold. Only one positive cast was made from each negative mold to maximize surface structure fidelity, resulting in one replicast per berry template. Flat PDMS replicasts (no surface topography) were generated using glass microscope slides and were included as controls in all experiments.

### 2.2 Microscopic validation of PDMS replicasts

PDMS replicasts of berry surfaces were viewed using 4×, 10×, and 20× objectives (Olympus UPlanFLN) with an inverted optical microscope (IX71, Olympus, Center Valley, PA). Images were captured using an affixed ORCA-ER digital camera (Hamamatsu, Japan) and were analyzed using Metamorph imaging software (version 7.7.2.0, Universal Imaging Corporation). Fresh berries were observed using light microscopy with the same magnification. Images of fresh berries were compared to those of PDMS replicasts to ensure comparability in surface macro- and micro-scale topography. High-resolution replication of various structures was visually observed in replicasts and compared to fresh samples to ensure fidelity.

### 2.3 Virus propagation and quantification

To propagate Tulane virus (TV) (obtained from Dr. Jianrong Li, Ohio State University), confluent monolayers of the monkey kidney cell line MK2-LLC (ATCC® no. CCL-7™, Manassas, VA, U.S.) were cultured. MK2-LLC cells were cultured in reduced serum Minimum Essential Medium (Opti-MEM™, Gibco™, Thermo Fisher Scientific, Waltham, MA, U.S.), supplemented with 2% fetal bovine serum (FBS, GenClone™, Genesee Scientific, Morrisville, NC, U.S.) at 37 °C under a 5% CO_2_ atmosphere. To make a TV stock for experiments, MK2-LLC cells were washed with Hank’s Balanced Salt Solution (HBSS, Gibco™, Thermo Fisher Scientific, Waltham, MA, U.S.) and subsequently infected with TV at an MOI of 0.1. After a 1-h incubation at 37 °C, 15 mL of Opti-MEM with 2% FBS was added. The virus was harvested 48 h post-inoculation and subjected to three freeze–thaw cycles, followed by centrifugation at 3,000 rpm for 20 min at 4 °C. Viral stock was collected and stored at −80 °C until use.

Plaque assays were performed in LLC-MK2 cells to quantify TV. Briefly, cells were seeded into six-well plates (Corning Life Sciences, Wilkes-Barre, PA) at a density of 10^6^ cells per well. After 24 h of incubation at 37 °C, MK2-LLC cell monolayers were infected with 400 μL of a 10-fold dilution series of TV. Plates were then incubated for 1 h at 37 °C with gentle agitation every 15 min. Each well was overlaid with 2.5 mL of Dulbecco’s Modified Eagle Medium (DMEM, Gibco™, Thermo Fisher Scientific, Waltham, MA, U.S.) containing 1% agarose (UltraPure™ LMP Agarose, Invitrogen, Waltham, MA, U.S.), 2% FBS, and Penicillin–Streptomycin (Gibco™, Thermo Fisher Scientific, Waltham, MA, U.S.). After incubation at 37 °C and 5% CO_2_ for 2 days, the plates were fixed with 10% formaldehyde, and the overlay was removed. The plaques were visualized by staining with crystal violet (0.05% w/v). Viral titer was expressed as mean log_10_ plaque-forming unit (PFU)/mL ± standard deviation.

### 2.4 TV inoculation and recovery of fresh berry and berry PDMS replicast surfaces

Half-inch diameter coupons of the surfaces of fresh blueberries, strawberries, and raspberries were cut using a cork borer. The same procedure was used to create coupons of replicasts for each berry. TV stock was prepared as described above to achieve a titer of 1 × 10^7^ PFU/mL. The inoculum used on strawberry and raspberry replicasts was supplemented with 0.05% TWEEN® 20 (Sigma-Aldrich, St. Louis, MO, U.S.) to reduce surface hydrophobicity to comparable levels to that of fresh berries. No surfactant was added to the viral inoculum used on replicasts of blueberries. Coupons of replicasts and fresh berries were placed in a biosafety cabinet and spot-inoculated with 50 μL of virus stock to achieve a titer of 2 × 10^5^ PFU per coupon. The inoculated coupons were air-dried in the biosafety cabinet for 1 h prior to sanitizer treatment.

**Figure 1:**
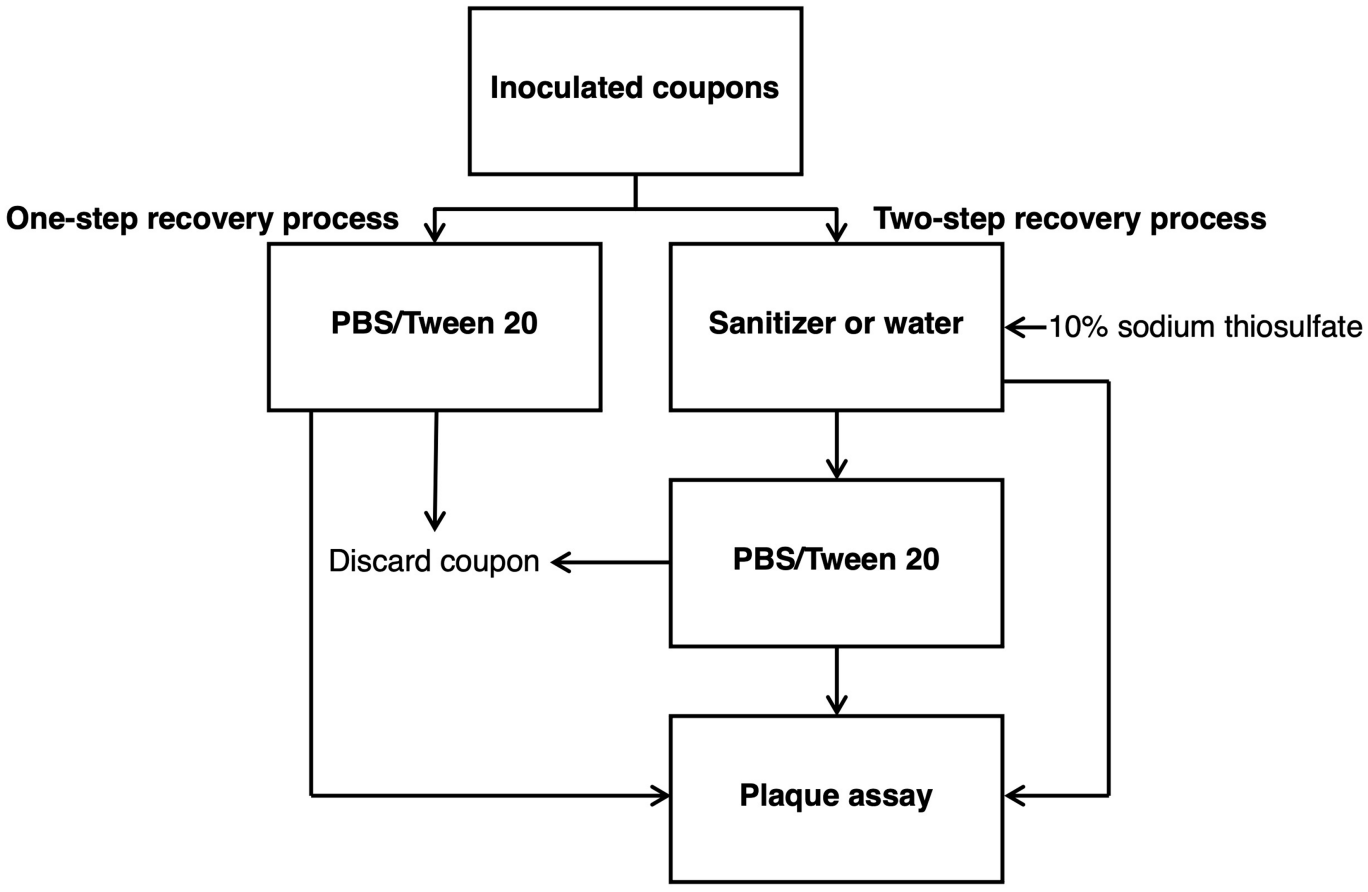
Flowchart of experimental design for one-step and two-step recovery procedures. In the one-step recovery procedure, TV-inoculated fresh and replicast berry surface coupons were transferred directly to PBS with 0.05% Tween 20 solution and agitated for 30 s before coupon removal. Plaque assays were performed on the PBS with 0.05% Tween 20 solution. In the two-step recovery procedure, TV-inoculated coupons were first transferred to water (control) or sanitizer (SH or PAA) and agitated for 30 s before 10% sodium thiosulfate was added. Coupons were then transferred to PBS with 0.05% Tween 20 solution and agitated for 30 s before coupon removal. Plaque assays were performed on the water or sanitizer solution and the PBS/Tween 20 solution.

To determine viral recovery from replicasts and fresh berry tissue, a previously described procedure was utilized (Figure 1) (Lightbown and DiCaprio, 2025). Inoculated fresh and replicast coupons were transferred to a 15-mL conical tube containing 1 mL PBS supplemented with 0.05% Tween 20, and the virus titer was determined in the solution by viral plaque assay (one-step recovery procedure). For mock sanitizer-treated samples, after 1 h incubation, inoculated coupons were transferred to 15-mL conical tubes containing 1 mL Milli-Q water to mimic the procedure to be used during sanitizer treatment experiments (control two-step recovery procedure). Coupons were agitated in sanitizer solution for 30 s using a vortex mixer, and then 10 μL of 10% sodium thiosulfate (Sigma-Aldrich, St. Louis, MO, U.S.) was added to each tube. The residual solution was retained for viral enumeration by plaque assay. Coupons were then transferred to a 15-mL conical tube containing PBS with 0.05% Tween 20. Coupons were agitated in rinse solution for 30 s, and the virus titer in wash solutions was determined by plaque assay. Each treatment included three replicates, and the virus titer is expressed as the mean log_10_ PFU/mL ± 1 standard deviation. Recovery efficiencies for the one-step and two-step recovery procedures were calculated by dividing the log_10_ PFU/mL of recovered virus by the log_10_ PFU/mL value of the initial inoculum per coupon and multiplying by 100 to get the percent recovery.

### 2.5 Sanitizer inactivation of TV on PDMS replicasts and fresh berries

Sodium hypochlorite (SH) (5% available chlorine, Spectrum Chemical Mfg. Corp., New Brunswick, NJ, U.S.) was diluted in Milli-Q water to achieve treatment concentrations of 50 and 200 ppm. The concentration of free chlorine was determined using the ColorQ® High Range Chlorine kit (LaMotte Company, Chestertown, MD, U.S.), and the pH was adjusted to 7.0. Peracetic acid (PAA) (3.5% w/w, RICCA Chemical Company, Arlington, TX, U.S.) was diluted in Milli-Q water to achieve treatment concentrations of 20 ppm and 80 ppm. The concentration of PAA was determined using the hydrogen peroxide and peracetic acid kit (LaMotte Company, Chestertown, MD, U.S.). Once concentration was verified, each concentration of sanitizer was transferred to a 15-mL sterile conical tube.

For sanitizer inactivation, a previously described procedure was utilized (Figure 1) (Lightbown and DiCaprio, 2025). After 1 h of incubation, inoculated coupons were transferred to 15-mL conical tubes containing 1 mL of sanitizer. Coupons were agitated in sanitizer solution for 30 s using a vortex mixer, and then 10 μL of 10% sodium thiosulfate (Sigma-Aldrich, St. Louis, MO, U.S.) was added to each tube to neutralize residual sanitizer. Sanitizer solutions were retained for viral enumeration by plaque assay. Following sanitizer treatment, coupons were transferred to a 15-mL conical tube containing PBS with 0.05% Tween 20. Coupons were agitated in rinse solution for 30 s. The virus titer in both collected solutions was determined by plaque assay. Each treatment included three replicates, and the virus titer is expressed as the mean log_10_ PFU/mL ± 1 standard deviation.

### 2.6 Plaque assay of sanitizer and rinse solutions

Plaque assays on the collected sanitizer and rinse solutions were performed to evaluate the efficacy of sanitizers on viral inactivation. To prepare plates for plaque assay, 2 mL of confluent MK2 cells were added to each well of a six-well tissue culture plate and incubated for 24 h. Serial dilutions were performed on both sanitizer and rinse solutions. Excess media was removed from the wells of the plates, and 400 μL of the appropriate serial dilution was added to each corresponding well. Plates were allowed to incubate at 37 °C for 1 h before the addition of agarose overlay solution. Plates were then allowed to solidify before incubation at 37 °C for 48 h. Plates were then fixed with 10% formaldehyde solution for 2 h and then stained with 1% crystal violet in 15% ethanol solution overnight. Plates were rinsed, and plaques were counted to enumerate recovered infectious TV titer in plaque-forming units (PFU/mL).

### 2.7 Statistical analysis

Statistical analysis was performed using Microsoft Excel using the *t*-test, paired two-sample t-test for means, to evaluate differences in recovery efficiency for replicasts and fresh berries. All sanitizer experiments were performed in triplicate for both replicast and fresh berry samples. Statistical analysis was performed using RStudio with R version 4.3.2 (The R Foundation for Statistical Computing, Vienna, Austria) to calculate one-way analysis of variance (ANOVA) and estimated marginal means (EMMs) between different sanitizers and types of berries. A *p*-value <0.05 was considered to be significant, and *p*-values were adjusted *post-hoc* using the Tukey HSD method.

## 3 Results

### 3.1 Comparison of virus recovery from replicasts and fresh berries using single or multi-step recovery process

**Figure 2:**
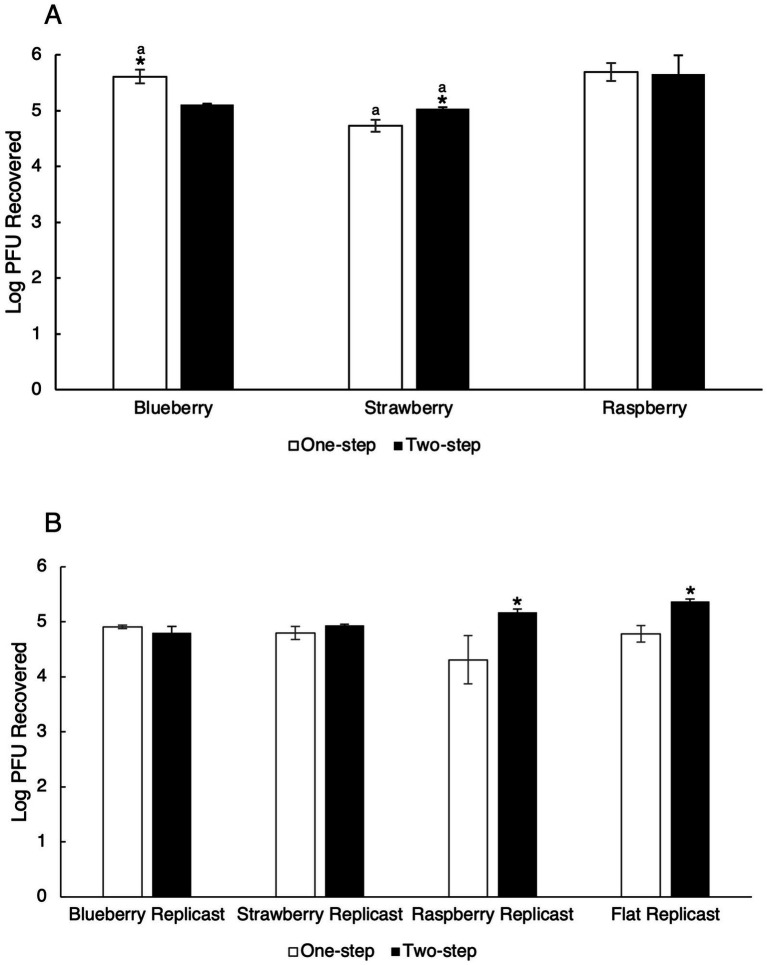
Comparison of TV recovery from surface-inoculated blueberry, strawberry, and raspberry and berry replicasts using a one-step or two-step recovery procedure. **(A)** Fresh berries and **(B)** Replicast berries. “One-step” indicates TV-inoculated coupons were transferred directly to PBS/Tween 20 solution in a one-step recovery procedure. “Two-step” indicates coupons were first transferred to Milli-Q water and then to PBS/Tween 20 solution in a two-step recovery procedure. Log_10_ PFU recovered represents the log-transformed Tulane virus titer recovered from coupons after the washing procedure. Analysis of variance (ANOVA) with the Tukey HSD method *post-hoc* applied to the data to determine significance. “*” Denotes a significantly higher viral recovery (*p*-value <0.05) comparing one-step wash and two-step wash. “a” Denotes a significantly higher viral recovery (*p*-value <0.05) comparing fresh and replicast samples in 1A and 1B.

Blueberries inoculated with TV that were directly transferred to the PBS + 0.05% Tween 20 recovery solutions (one-step recovery) had a level of virus recovery of 5.61 log_10_ PFU/mL (Figure 2A). Blueberry replicasts inoculated with TV and subjected to the one-step recovery process had 4.91 log_10_ PFU/mL recovered (Figure 2B). Strawberries and strawberry replicasts inoculated with TV and processed via the one-step recovery procedure had recovered viral titers of 4.73 and 4.80 log_10_ PFU/mL, respectively. The one-step recovery process led to the detection of 5.69 log_10_ PFU/ml from fresh raspberries and 4.31 log_10_ PFU/mL detected from raspberry replicasts. There was a significant difference found between fresh and replicast blueberry and strawberry surfaces, with the one-step recovery procedure resulting in higher recovery from fresh berries compared to replicasts (*p*-values of 0.015 and 0.017), but no significant difference in recovery between fresh and replicast raspberry surfaces using the same procedure.

For the mock sanitizer control samples, where TV-inoculated coupons were transferred to Milli-Q water prior to the wash solution (two-step recovery process), the recovery of TV for fresh blueberries was 5.11 log_10_ PFU/mL and 4.80 log_10_ PFU/mL for replicast blueberries (Figures 2A,B). The recovery levels of TV using the two-step process for fresh strawberries were 5.04 log_10_ PFU/mL, and from replicast strawberries were 4.93 1og_10_ PFU/mL. Finally, for fresh raspberries, the TV recovery level using the two-step process was 5.66 log_10_ PFU/mL and 5.17 log_10_ PFU/mL for raspberry replicasts. Using this two-step recovery process, a significant difference between fresh and replicast surfaces was only found in strawberries (*p*-value 0.008). No significant differences were seen between fresh or replicast surfaces of blueberry or raspberry with the two-step recovery process.

Recovery efficiencies were calculated to compare the control one-step recovery procedure and the control two-step recovery procedure in recovering infectious virus from fresh and replicast berries. Using 5.33 log_10_ PFU/coupon as the baseline, the recovery efficiency percentage was determined from the average viral titer recovered from the berry coupon after the washing procedure (Table 1). The two-step recovery process decreased efficiency in the recovery of TV from blueberries and blueberry replicasts as compared to the one-step recovery process. The opposite effect was observed in fresh and replicast strawberries and raspberries. Utilizing the two-step recovery process increased recovery of TV from fresh strawberries, replicast strawberries, and replicast raspberries. Flat PDMS replicast controls also showed increased recovery efficiency utilizing the two-step recovery procedure (100.8%) as compared to the one-step recovery procedure (89.7%).

**Table 1:** Recovery efficiencies for fresh and replicast blueberry, strawberry, and raspberry surfaces after a one-step and two-step recovery procedure.

Recovery efficiency (%)						
Surface	Surface type	One wash	Two wash	Difference (One vs. Two)	Difference (Fresh vs. Replicast)	
					One wash	Two wash
Blueberry	Fresh	105.3 ± 2.3	96.0 ± 0.21	−9.33	13.2	5.86
	Replicast	92.1 ± 0.53	90.1 ± 2.2	−2.02		
Strawberry	Fresh	88.8 ± 2.0	94.6 ± 0.39	5.81	1.22	2.04
	Replicast	90.0 ± 2.3	92.6 ± 0.42	2.55		
Raspberry	Fresh	106.5 ± 2.8	106.2 ± 6.3	−0.325	25.6	9.04
	Replicast	80.9 ± 8.2	97.1 ± 1.1	16.3		
Flat	Replicast	89.7 ± 2.8	100.8 ± 0.77	11.1	–	

“Flat” indicates control replicast without surface topography. “One wash” and “Two wash” columns indicate recovery efficiency from fresh or replicast berries after the control one-step recovery procedure or control two-step recovery procedure, respectively, with the “Difference (One vs. Two)” column indicating the difference in percent recovery between the one-step and two-step recovery procedure. The “Difference (Fresh vs. Replicast) column” indicates the difference in percent recovery between the fresh and replicast berry after the two washing procedures. Standard deviation values for all replicates are represented after “±”.

### 3.2 Efficacy of SH and PAA on inactivation of TV on fresh berries and replicasts

**Figure 3:**
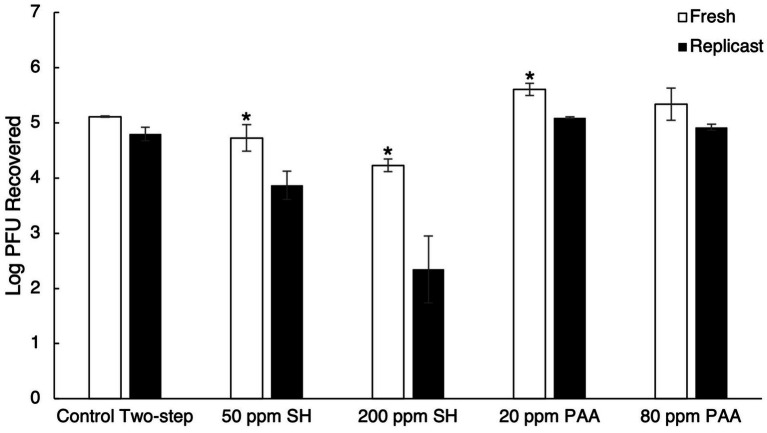
Comparison of TV recovery from blueberry replicasts to fresh blueberries after sanitizer treatment. The process includes a two-step control washing procedure, treatment with 50 ppm SH, treatment with 200 ppm SH, treatment with 20 ppm PAA, and treatment with 80 ppm PAA. Log_10_ PFU recovered represents log-transformed Tulane virus titer recovered from coupons after a two-step washing procedure. Analysis of variance (ANOVA) with the Tukey HSD method *post-hoc* applied to the data to determine significance. “*” Denotes a significantly higher viral recovery (*p*-value < 0.05) comparing fresh and replicast samples.

Due to instances of increased viral recovery from berry surfaces and more comparable recovery between fresh and replicast surfaces with the control two-step recovery procedure, the two-step process was adopted for sanitizer inactivation trials. The two-step recovery control fresh berry and replicast samples (mock sanitizer control samples) were used as the baseline to determine viral inactivation by sanitizer treatment. With the low concentration treatment of SH (50 ppm), a 0.38 log_10_ PFU reduction in fresh blueberries and a 0.93 log_10_ PFU reduction in replicast blueberries were observed (Figure 3). A higher concentration treatment of SH (200 ppm) resulted in a 0.88 log_10_ PFU reduction in fresh blueberries and a 2.45 log_10_ PFU reduction in replicast blueberries (Figure 3). Within the PAA treatment groups, there were instances of a higher level of virus recovery than in the mock-sanitizer control samples. With the low concentration of PAA (20 ppm) treatment, there was a 0.50 log_10_ PFU and 0.29 log_10_ PFU greater virus recovery in fresh and replicast blueberries, respectively, compared to the control (Figure 3). A 0.23 log_10_ PFU and 0.12 log_10_ PFU increase in recovered virus was seen in fresh and replicast blueberries, respectively, as compared to the control with the 80 ppm PAA treatment (Figure 3).

**Figure 4:**
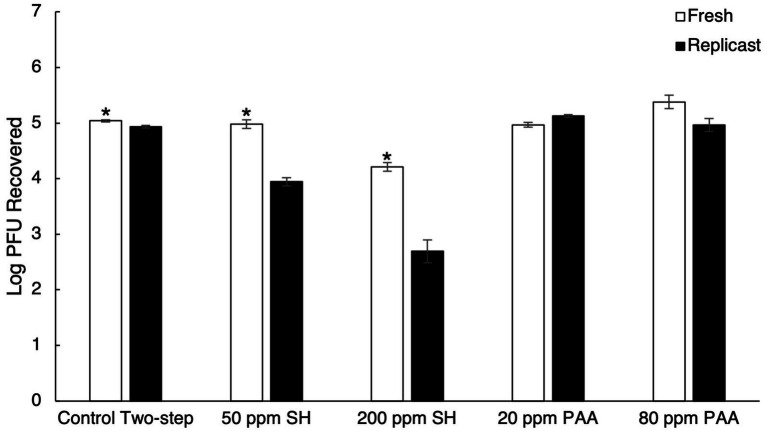
Comparison of TV recovery from strawberry replicasts to fresh strawberries after sanitizer treatment. It includes a control two-step washing procedure, treatment with 50 ppm SH, treatment with 200 ppm SH, treatment with 20 ppm PAA, and treatment with 80 ppm PAA. Log_10_ PFU recovered represents log-transformed Tulane virus titer recovered from coupons after a two-step washing procedure. Analysis of variance (ANOVA) with the Tukey HSD method *post-hoc* applied to the data to determine significance. “*” Denotes a significantly higher viral recovery (*p*-value < 0.05) comparing fresh and replicast samples.

Treatment with 50 ppm SH led to a 0.06 log_10_ PFU reduction in viral titer on fresh strawberries and a 0.98 log_10_ PFU reduction in viral titer on replicast strawberries (Figure 4). A 0.83 log_10_ PFU reduction in viral titer was observed with treatment of fresh strawberries with 200 ppm SH, and a 2.24 log_10_ PFU reduction in replicast strawberries occurred with the 200 ppm SH treatment (Figure 4). The higher concentration of PAA (80 ppm) also displayed greater TV recovery in some strawberry samples, similar to the observation in blueberries. In fresh strawberries, 20 ppm PAA treatment led to a 0.07 log_10_ reduction, while the same treatment led to a 0.20 log_10_ increase in recovered TV in replicast strawberries compared to the control (Figure 4).

**Figure 5:**
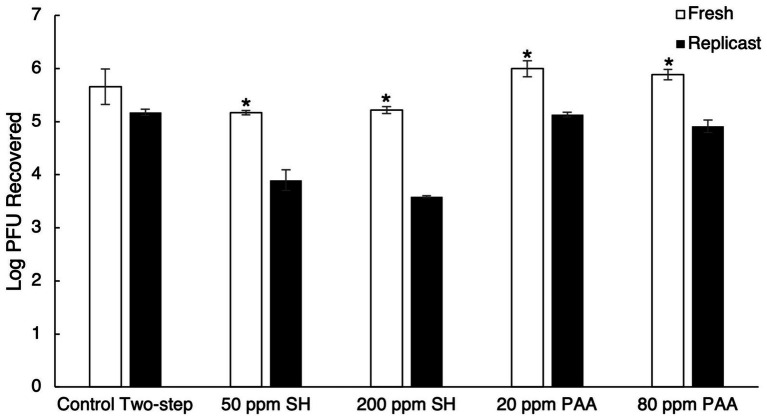
Comparison of TV recovery from raspberry replicasts to fresh raspberry after sanitizer treatment. It includes a control two-step washing procedure, treatment with 50 ppm SH, treatment with 200 ppm SH, treatment with 20 ppm PAA, and treatment with 80 ppm PAA. Log_10_ PFU recovered represents log-transformed Tulane virus titer recovered from coupons after a two-step washing procedure. Analysis of variance (ANOVA) with Tukey HSD method *post-hoc* applied to data to determine significance. “*” Denotes a significantly higher viral recovery (*p*-value < 0.05) comparing fresh and replicast samples.

In raspberries treated with 50 ppm SH, a 0.49 log_10_ PFU reduction in viral titer and a 1.27 log_10_ PFU reduction were observed for fresh and replicast samples, respectively (Figure 5). Treatment with 200 ppm SH led to a 0.22 log_10_ PFU reduction in viral titer in fresh raspberries and a 1.59 log_10_ PFU reduction in replicast raspberries. In raspberries, the 20 ppm PAA treatment group displayed higher recovery of TV from fresh raspberries as compared to the control (a 0.23 log_10_ increase) and a reduction in virus recovery from replicast raspberries compared to the control (a 0.26 log_10_ decrease) (Figure 5).

The higher concentration of SH (200 ppm) led to a greater reduction in infectious TV compared to the lower concentration for all fresh and replicast berry samples, apart from fresh raspberries. However, the difference in log_10_ reduction was not significant in fresh raspberries between the two SH treatments (*p*-value of 0.997). Significantly less virus was recovered in replicast samples than in the fresh samples for all three berries in both SH treatment groups. Neither concentration of PAA was effective at reducing infectious viral titer by more than 0.5 log_10_ in either fresh or replicast berries and actually improved viral recovery from berries in some cases.

### 3.3 Impact of surface topography on virus recovery and inactivation

**Figure 6:**
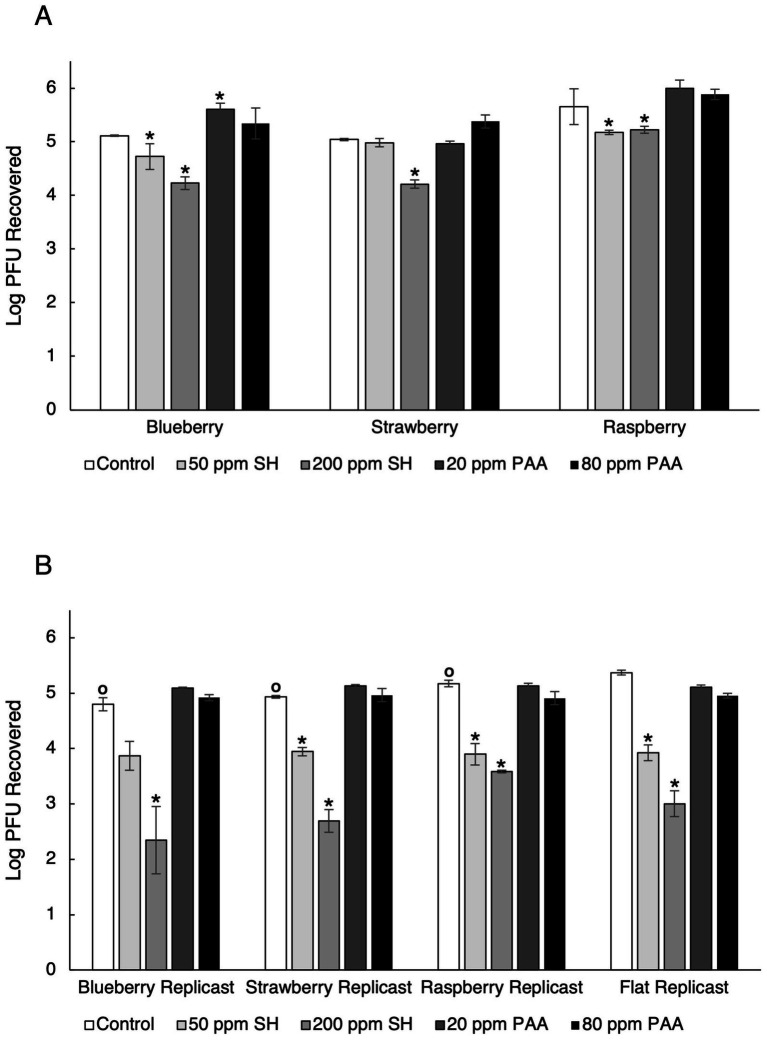
TV recovery from fresh berries and replicast berries after a two-step recovery procedure. **(A)** Fresh berries **(B)** Replicast berries. Log_10_ PFU recovered represents log-transformed Tulane virus titer recovered from coupons after a two-step washing procedure. Analysis of variance (ANOVA) with the Tukey HSD method *post-hoc* applied to the data to determine significance. “*” Denotes a significant difference (*p*-value < 0.05) between sanitizer treatment and respective control. “○” Denotes a significant difference (*p*-value < 0.05) between flat control and berry control.

PDMS coupons with no surface topography (flat) controls had a level of viral recovery of 5.37 log_10_ PFU/coupon in the control two-step recovery procedure. In the control, viral recovery from berry replicasts was significantly lower than that from flat replicasts for all three berries (*p*-values of 0.007, 0.002, and 0.003 for blueberry, strawberry, and raspberry replicasts, respectively) (Figure 6B). No significant differences were found between flat and blueberry or strawberry replicasts for any sanitizer treatment tested. However, there was a significant difference found between flat and raspberry replicasts in the higher SH treatment (200 ppm) (*p*-value = 0.044) and the lower PAA treatment (20 ppm) (*p*-value = 0.025). With flat replicasts, no significant differences were found between the control group and either PAA treatment concentration. Significant differences were found between the control group and both SH treatment concentrations, as well as between the two SH concentrations tested.

## 4 Discussion

To evaluate the influence of berry surface topography on viral recovery and inactivation, both washing strategy and chemical sanitizer inactivation were incorporated into this study. Replicasts of blueberries, strawberries, and raspberries were fabricated to mimic the range in microscale surface topography of fresh berries. These replicasts and their fresh counterparts were then subjected to a one- and two-step washing procedure to determine viral recovery efficiency. Washing procedure trials were performed to find an effective washing strategy to maximize viral recovery from berry surfaces. Sanitizer trials were then conducted using the two-step washing procedure developed, where two concentrations of both SH and PAA were tested on TV surface-inoculated fresh and replicast blueberries, strawberries, and raspberries to elucidate the impact of berry surface topography on viral inactivation by chemical sanitizers.

Before beginning experiments on the efficacy of chemical sanitizers, it was necessary to adjust the hydrophobicity of some berry replicasts in order to make the contact angle comparable to that of their fresh berry counterparts. The material used to fabricate replicasts, PDMS, is an inherently hydrophobic material, meaning it has a contact angle of greater than 90° that can, depending on the surface roughness, reach up to 180°. A lower contact angle leads to improved surface wettability, allowing for greater contact between the inoculum and the plant surface. Previous studies have shown that the contact angle of fresh blueberry cuticle is high, measuring 127.1° and 131.3° in two separate studies (Ramírez et al., 2020; Velásquez et al., 2011). In contrast, the measured contact angle of fresh strawberries has been reported as 74.8°, a far lower value than that of fresh blueberries (Velásquez et al., 2011). The hydrophilic surface of fresh strawberries leads to greater surface wettability compared to fresh blueberries. It has been proposed that fruit epicarp characteristics are determined by the changes in the ovarian region involved in fruit development (Velásquez et al., 2011). As both strawberries and raspberries are aggregate fruits, meaning they develop from multiple ovaries of a single flower, they likely have similar epicuticular wax chemical compositions and structures. Blueberries develop from a single ovary of a single flower and therefore have differing cuticle characteristics from aggregate fruits. A previous study has shown that the contact angle of flat PDMS replicasts is 107.5°, and that the addition of the surfactant Tween 20 can reduce the hydrophobicity to comparable levels of fresh plant tissue (74.4°) (Lightbown and DiCaprio, 2025). Based on the need to reduce surface hydrophobicity of PDMS replicasts of strawberries and raspberries to more accurately represent the surface hydrophobicity of the representative fresh berry, all viral inoculum applied to PDMS replicasts of strawberry and raspberry surfaces was supplemented with 0.05% Tween 20. As contact angle measurements for fresh raspberries have not been reported on, similar contact angles between aggregate fruit surfaces, strawberry and raspberry, were targeted. Viral inoculum applied to blueberry surfaces was not supplemented with a surfactant, as the contact angle of PDMS replicasts is already close to that of fresh blueberry cuticle.

It was found that a one-step washing procedure led to significant differences in viral recovery between fresh and replicast samples of blueberries and strawberries (Table 1). Adopting a two-step washing procedure by adding a mock sanitization step only made viral recovery comparable (no significant difference) between fresh and replicast blueberry and raspberry surfaces. The two-step washing procedure did, however, increase viral recovery from both fresh and replicast strawberry surfaces compared to the one-step wash. The average recovery efficiency increased by 5.8 and 2.6% in fresh and replicast strawberries, respectively, with the extra wash. Raspberry replicasts exhibited a 16.3% increase in recovery efficiency with the two-step washing procedure compared to the one-step. Interestingly, both fresh and replicast blueberries exhibited a decrease in recovery efficiency with increasing washes (2.02 and 9.33%, respectively), yet the decrease was only found to be significant in fresh blueberries. This could be due to the removal of the protective cuticular wax covering the blueberry epicarp through more extended washing procedures. With the removal of this wax, leaching of antiviral compounds could occur from the blueberry interior (Bozkurt et al., 2021), reducing the recovery of infectious virus. Overall, the two-step washing procedure generally improved virus recovery from samples and reduced the contrast in recovery between fresh and replicast berries compared to the one-step washing procedure, emphasizing the importance of a multi-step washing process in berries.

In general, surface topography of berry replicasts had a significant impact on viral recovery. Compared to control replicasts without any surface topography, all three berry types had a significantly lower recovery of infectious virus in the control two-step washing procedure without any chemical sanitizer addition. This means that the surface topography of these berry replicasts allowed for more infectious virus to remain on the replicasts even after washing. Flat replicasts demonstrated up to a 0.57 log_10_ greater recovery of infectious virus as compared to the recovery of virus from berry replicasts (Figure 6B). This aligns with a previously published study that washing with water is ineffective at reducing HuNoV titer by more than 1.5 log_10_ (Butot et al., 2008). As blueberries are classified as a “true” berry with a smooth surface, and strawberries and raspberries are aggregate berries with an irregular surface (Mitcham, 2007), it would be predicted that the more textured, irregular surface of aggregate fruits would provide greater opportunity for viral protection from washing due to more surface structures that allow for harborage of viral particles as compared to true berries (Bozkurt et al., 2021). The viral detection efficiency from aggregate berries is also lower than that of true berries, such as blueberries, as the virus is easier to remove from smooth skin and blueberries have a cuticular wax that can prevent the leaching of antiviral compounds from the berry flesh (Bozkurt et al., 2021). The high contact angle of fresh blueberries could contribute to this finding, as high surface hydrophobicity has been shown to have a negative impact on bacterial adhesion, with more hydrophobic surfaces experiencing less bacterial adhesion than hydrophilic surfaces (Tian et al., 2025). Similarly to fresh berries, a greater degree of surface roughness on other silicone-based material surfaces has been linked to greater adhesion of bacteria to those surfaces, though this effect has yet to be demonstrated for viruses (Tang et al., 2009).

However, a greater recovery efficiency of the virus was seen in strawberry and raspberry replicasts than in blueberry. A similar result was observed in the extraction efficiency of the enteric virus Mengovirus from frozen blueberries, strawberries, and raspberries, with a 5.4, 9.9, and 16.3% recovery efficiency recorded, respectively (Butot et al., 2009). This could be because the styles, or hair-like projections, of the aggregate berries are not preserved during PDMS casting or are damaged during freezing. These styles on raspberries have been shown to be responsible for greater retention of pathogens by raspberries compared to blueberries (Kniel et al., 2002). In previous study, HuNoV VLPs have demonstrated binding capacity to exposed carbohydrate moieties found on lettuce cell walls (Esseili et al., 2012; Gao et al., 2016). It is possible that the styles found on aggregate fruit surfaces contain similar carbohydrate moieties that allow for virus adhesion. This could also contribute to why there was greater viral recovery from fresh strawberries and raspberries than their replicast counterparts in the control group and all sanitizer treatment groups. A significant difference in viral recovery between fresh blueberry and fresh raspberry in the control group and in both sanitizers at every concentration tested shows that the type of berry does have a marked impact on viral recovery.

Previous studies have been conducted to characterize the inactivation of foodborne viruses using SH and PAA, two chemical sanitizers commonly used in the produce industry pre- and post-harvest. The concentration of chlorine that can be used in contact with fresh edible produce, that is, in wash water, is limited to 200 ppm, while PAA concentration is limited to 80 ppm. These chemical sanitizers have been shown to be effective at reducing or eliminating bacterial pathogens at these recommended concentrations; however, they are notably less effective at reducing infectious virus quantities (Chiu et al., 2015; Cromeans et al., 2014). Plaque assays were used to quantify infectious virus inactivation by sanitizers in this study since real-time RT-PCR analysis methods cannot distinguish between infectious and non-infectious viral particles. All chemical sanitizers employed in this study resulted in the recovery of infectious virus after treatment.

The maximum concentration of chlorine allowed on fresh edible produce (200 ppm) has previously been shown to be effective at decreasing HuNoV GII viral load on blueberries, strawberries, and raspberries by 3.0, 1.4, and 0.9 log_10_ units, respectively, with a 30-s contact time, though this was determined by real-time RT PCR (Butot et al., 2008). Less than a 1 log_10_ reduction in infectious virus was seen in strawberries and raspberries contaminated with a HuNoV surrogate (MNV-1) after treatment with 200 ppm chlorine for 2 min (Predmore and Li, 2011). Soaking fresh blueberries in 100 ppm SH for 1 min, however, led to a 4.2 log_10_ reduction in infectious MNV-1 (Takahashi et al., 2018). Washing fresh raspberries in 50 ppm SH solution only reduced infectious MNV-1 by 1.2 log_10_ after 20 s (Maks et al., 2019). Using the bacteriophage MS2 as a surrogate for HuNoV, less than a 1.5 log_10_ reduction in infectious phage was observed with a 100 ppm SH treatment of contaminated strawberries for 5 min (Dawson et al., 2005) and a 200 ppm SH treatment of contaminated strawberries for 12 s in an industrial-scale washing unit (Casteel et al., 2009). In this study, significant differences between fresh and replicast berries throughout the chemical sanitizer treatments were found, especially in the SH treatment groups. All three types of berry replicasts experienced greater reduction in viral titer than their fresh counterparts with SH treatment, with less infectious virus recovered for each (Figure 6A).

Organic material present in the wash water is able to react with chlorine to reduce the sanitizer’s efficacy by decreasing the amount of active or “free” chlorine available (Fuzawa et al., 2019). The discrepancies between fresh and replicast can be attributed to the organic material present in fresh berries that is not a factor in replicast berries. The high concentration of SH was able to reduce infectious virus by 0.22–0.88 log_10_ in fresh berries and 1.59–2.45 log_10_ in replicast berries, with blueberries experiencing the greatest reduction and raspberries experiencing the least reduction in viral titer in both fresh and replicast groups (Figure 6). Fresh blueberries also experienced the greatest viral reduction of the three berries, with the low concentration of SH leading to an approximate 0.5 log_10_ reduction in blueberries (Figure 3). This agrees with previous study showing that the virus on blueberries is typically more susceptible to SH treatment than strawberries or raspberries, and SH is typically not effective at reducing infectious virus on berries by more than 1–2 log_10_. In this study, it is proposed that the reduced efficacy of SH treatment on fresh berries compared to their replicasts is due to the organic material found on fresh berries.

The efficacy of PAA is not influenced by organic material present, yet it has also been shown to have limited efficacy on HuNoV surrogates and other foodborne viruses (Fuzawa et al., 2020; Hirneisen and Kniel, 2013). Other study on PAA inactivation of HuNoV in berries has shown that PAA at the maximum concentration allowed for edible produce contact (80 ppm) reduced infectious MNV-1 on strawberries by less than 2 log_10_ after a 2-min contact time (Ortiz-Solà et al., 2020). Washing fresh raspberries with an 80 ppm PAA solution only led to a 1.6 log_10_ reduction in infectious MNV-1 after 20 s (Maks et al., 2019). An 80 ppm PAA treatment for 1 min did, however, lead to a 2.5–3 log_10_ reduction in fresh blueberries contaminated with MNV-1 (Leblanc et al., 2021). While both concentrations of SH treatment resulted in some reduction of infectious virus in this inactivation study, neither PAA treatment led to any significant reduction in virus recovery. This could be attributed to the difference in disinfection mechanisms utilized by these two sanitizers. While both sanitizers damage viral capsid proteins, thereby impacting host receptor binding, PAA has been shown to cause aggregation of TV particles in solution due to its low pH, improving viral resistance to disinfection (Fuzawa et al., 2020). This could explain why recovered solutions from both fresh and replicast samples demonstrated little reduction in infectious virus with PAA treatment. The low pH of PAA, combined with the naturally low pH of berry surfaces, could also help explain why there were some instances of improved recovery in the PAA treatment groups compared to the control. Viral aggregation might not only improve disinfection resistance but also improve the detection of the virus if there are viral agglomerates in the recovered solution. This study supports that commonly available commercial sanitizers are ineffective at completely inactivating viral pathogens of concern in the produce industry, aligning with data previously published on the limited impact of chemical sanitizers on HuNoV surrogates.

In summary, PDMS casting of berries is a viable method to aid in viral recovery studies with consistent and replicable results. Berry replicasts should be used with caution in viral inactivation studies to prevent overestimation of sanitizer efficacy. While physical berry characteristics may have some impact on HuNoV surrogate inactivation and removal, it is likely that other factors, particularly specific and non-specific binding mechanisms, are also at play. This has been found to be especially true in raspberries, with their resistance to disinfection and high standard deviations due to their complex surface structure. Though PDMS replicasts have inherent limitations, as the chemical and biological compositions of fresh berries are not preserved with casting, further modifying PDMS replicasts to be more representative of plant-specific or environmental conditions could be valuable to help determine underlying mechanisms for viral persistence. In addition to the hydrophobicity modifications incorporated in this study, this could be further examined by modulating electrostatic characteristics or introducing native plant microbiota to replicasts. Human norovirus has been associated with berry-related foodborne outbreaks previously, yet the level of contamination leading to outbreaks has not been characterized. It could be of value in future studies to evaluate the inoculum level on recovery efficiency, now that it has been established that replicast berries are suitable for such study. The high fidelity and recovery efficiencies between fresh and replicast samples in the control group support that PDMS berry replicasts can serve as a useful model system to mimic foodborne pathogen deposition, adhesion, and removal from berry surfaces.

## Data Availability

The raw data supporting the conclusions of this article will be made available by the authors, without undue reservation.

## References

[ref1] Baert L. Debevere J. Uyttendaele M. (2009a). The efficacy of preservation methods to inactivate foodborne viruses. Int. J. Food Microbiol. 131, 83–94. doi: 10.1016/j.ijfoodmicro.2009.03.007, 19349089

[ref2] Baert L. Vandekinderen I. Devlieghere F. Van Coillie E. Debevere J. Uyttendaele M. (2009b). Efficacy of sodium hypochlorite and peroxyacetic acid to reduce murine norovirus 1, B40-8, listeria monocytogenes, and *Escherichia coli* O157:H7 on shredded iceberg lettuce and in residual wash water. J. Food Prot. 72, 1047–1054. doi: 10.4315/0362-028X-72.5.1047, 19517733

[ref3] Bosch A. Gkogka E. Le Guyader F. S. Loisy-Hamon F. Lee A. van Lieshout L. . (2018). Foodborne viruses: detection, risk assessment, and control options in food processing. Int. J. Food Microbiol. 285, 110–128. doi: 10.1016/j.ijfoodmicro.2018.06.001, 30075465 PMC7132524

[ref4] Bozkurt H. Phan-Thien K.-Y. van Ogtrop F. Bell T. McConchie R. (2021). Outbreaks, occurrence, and control of norovirus and hepatitis a virus contamination in berries: a review. Crit. Rev. Food Sci. Nutr. 61, 116–138. doi: 10.1080/10408398.2020.1719383, 32008374

[ref5] Butot S. Putallaz T. Amoroso R. Sánchez G. (2009). Inactivation of enteric viruses in minimally processed berries and herbs. Appl. Environ. Microbiol. 75, 4155–4161. doi: 10.1128/AEM.00182-09, 19395576 PMC2698352

[ref6] Butot S. Putallaz T. Sánchez G. (2008). Effects of sanitation, freezing and frozen storage on enteric viruses in berries and herbs. Int. J. Food Microbiol. 126, 30–35. doi: 10.1016/j.ijfoodmicro.2008.04.033, 18547667

[ref7] Casteel M. J. Schmidt C. E. Sobsey M. D. (2009). Chlorine inactivation of coliphage MS2 on strawberries by industrial-scale water washing units. J. Water Health 7, 244–250. doi: 10.2166/wh.2009.065, 19240350

[ref8] Chiu S. Skura B. Petric M. McIntyre L. Gamage B. Isaac-Renton J. (2015). Efficacy of common disinfectant/cleaning agents in inactivating murine norovirus and feline calicivirus as surrogate viruses for human norovirus. Am. J. Infect. Control 43, 1208–1212. doi: 10.1016/j.ajic.2015.06.021, 26254499

[ref9] Cromeans T. Park G. W. Costantini V. Lee D. Wang Q. Farkas T. . (2014). Comprehensive comparison of cultivable norovirus surrogates in response to different inactivation and disinfection treatments. Appl. Environ. Microbiol. 80, 5743–5751. doi: 10.1128/AEM.01532-14, 25015883 PMC4178592

[ref10] Dawson D. J. Paish A. Staffell L. M. Seymour I. J. Appleton H. (2005). Survival of viruses on fresh produce, using MS2 as a surrogate for norovirus. J. Appl. Microbiol. 98, 203–209. doi: 10.1111/j.1365-2672.2004.02439.x, 15610433

[ref11] Doan H. K. Antequera-Gómez M. L. Parikh A. N. Leveau J. H. J. (2020a). Leaf surface topography contributes to the ability of *Escherichia coli* on leafy greens to resist removal by washing, escape disinfection with chlorine, and disperse through splash. Front. Microbiol. 11:1485. doi: 10.3389/fmicb.2020.01485, 32765440 PMC7380079

[ref12] Doan H. K. Ngassam V. N. Gilmore S. F. Tecon R. Parikh A. N. Leveau J. H. J. (2020b). Topography-driven shape, spread, and retention of leaf surface water impacts microbial dispersion and activity in the Phyllosphere. Phytobiomes J. 4, 268–280. doi: 10.1094/PBIOMES-01-20-0006-R

[ref13] Esseili M. A. Gao X. Boley P. Hou Y. Saif L. J. Brewer-Jensen P. . (2019). Human norovirus histo-blood group antigen (HBGA) binding sites mediate the virus specific interactions with lettuce carbohydrates. Viruses 11:833. doi: 10.3390/v11090833, 31500340 PMC6784273

[ref14] Esseili M. A. Wang Q. Saif L. J. (2012). Binding of human GII.4 norovirus virus-like particles to carbohydrates of romaine lettuce leaf cell wall materials. Appl. Environ. Microbiol. 78, 786–794. doi: 10.1128/AEM.07081-11, 22138991 PMC3264112

[ref15] FAO and WHO (2024). “Microbiological risk assessment of viruses in foods. Part 1: food attribution, analytical methods and indicators- meeting report,” in Microbiological Risk Assessment Series (49), (Rome, Italy: FAO and WHO).

[ref16] FDA. (2025). Summary of FDA’S Strategy to Prevent Human Norovirus and Hepatitis A Outbreaks Associated with Fresh and Frozen Berries. U.S. Food & Drug Administration. Available online at: https://www.fda.gov/food/new-era-smarter-food-safety/summary-fdas-strategy-prevent-human-norovirus-and-hepatitis-outbreaks-associated-fresh-and-frozen (Accessed August 12, 2025).

[ref17] Fuzawa M. Araud E. Li J. Shisler J. L. Nguyen T. H. (2019). Free chlorine disinfection mechanisms of rotaviruses and human norovirus surrogate tulane virus attached to fresh produce surfaces. Environ. Sci. Technol. 53, 11999–12006. doi: 10.1021/acs.est.9b03461, 31517478

[ref18] Fuzawa M. Bai H. Shisler J. L. Nguyen T. H. (2020). The basis of Peracetic acid inactivation mechanisms for rotavirus and Tulane virus under conditions relevant for vegetable sanitation. Appl. Environ. Microbiol. 86:e01095-20. doi: 10.1128/AEM.01095-20, 32709728 PMC7499037

[ref19] Gao X. Esseili M. A. Lu Z. Saif L. J. Wang Q. (2016). Recognition of Histo-blood group antigen-like carbohydrates in lettuce by human GII.4 norovirus. Appl. Environ. Microbiol. 82, 2966–2974. doi: 10.1128/AEM.04096-15, 26969699 PMC4959087

[ref20] Hirneisen K. A. Kniel K. E. (2013). Norovirus surrogate survival on spinach during preharvest growth. Phytopathology 103, 389–394. doi: 10.1094/phyto-09-12-0231-fi, 23506365

[ref21] Jaykus L.-A. D’Souza D. H. Moe C. L. (2012). “Foodborne viral pathogens,” in Food Microbiology: Fundamentals and Frontiers, eds. Doyle M. P. Buchanan R. L. (Washington, DC, USA: ASM Press), 619–649.

[ref22] Kniel K. E. Lindsay D. S. Sumner S. S. Hackney C. R. Pierson M. D. Dubey J. P. (2002). Examination of attachment and survival of toxoplasma gondii oocysts on raspberries and blueberries. J. Parasitol. 88, 790–793. doi: 10.1645/0022-3395(2002)088[0790:EOAASO]2.0.CO;2, 12197133

[ref23] Koopmans M. Duizer E. (2004). Foodborne viruses: an emerging problem. Int. J. Food Microbiol. 90, 23–41. doi: 10.1016/S0168-1605(03)00169-7, 14672828 PMC7127053

[ref24] Leblanc D. Gagné M.-J. Brassard J. (2021). Effectiveness of water and sanitizer washing solutions for removing enteric viruses from blueberries. Food Control 126:108043. doi: 10.1016/j.foodcont.2021.108043

[ref25] Lightbown A. DiCaprio E. (2025). The role of produce surface topography on foodborne virus inactivation on polydimethylsiloxane topomimetic artificial leaf surfaces and fresh leafy green surfaces. Front. Plant Sci. 16:6467. doi: 10.3389/fpls.2025.1636467, 40949551 PMC12423083

[ref26] Maks N. Ye M. U. Swanson S. Lee A. Freeman B. B. Deng K. (2019). Evaluation of inactivating norovirus, hepatitis a, and *Listeria monocytogenes* on raspberries by sanitizer spray. J. Food Prot. 82, 869–877. doi: 10.4315/0362-028X.JFP-18-415, 31017811

[ref27] Mitcham E. (2007). “Quality of berries associated with preharvest and postharvest conditions,” in Berry Fruit: Value-Added Products for Health Promotion, ed. Zhao Y. (Boca Raton, USA: CRC Press), 219–240.

[ref28] Ortiz-Solà J. Abadias M. Colás-Medà P. Sánchez G. Bobo G. Viñas I. (2020). Evaluation of a sanitizing washing step with different chemical disinfectants for the strawberry processing industry. Int. J. Food Microbiol. 334:108810. doi: 10.1016/j.ijfoodmicro.2020.108810, 32805511

[ref29] Predmore A. Li J. (2011). Enhanced removal of a human norovirus surrogate from fresh vegetables and fruits by a combination of surfactants and sanitizers. Appl. Environ. Microbiol. 77, 4829–4838. doi: 10.1128/AEM.00174-11, 21622782 PMC3147408

[ref30] Ramírez L. Sáez C. Matiacevich S. (2020). New methodology to measure in vivo permeance on blueberry (*Vaccinium corymbosum*) skin: a correlation to quality during storage. Postharvest Biol. Technol. 161:110894. doi: 10.1016/j.postharvbio.2019.04.020

[ref31] Sun T. Lazouskaya V. Jin Y. (2019). Polydimethylsiloxane replicas efficacy for simulating fresh produce surfaces and application in mechanistic study of colloid retention. J. Food Sci. 84, 524–531. doi: 10.1111/1750-3841.14479, 30775789

[ref32] Takahashi M. Okakura Y. Takahashi H. Imamura M. Takeuchi A. Shidara H. . (2018). Heat-denatured lysozyme could be a novel disinfectant for reducing hepatitis a virus and murine norovirus on berry fruit. Int. J. Food Microbiol. 266, 104–108. doi: 10.1016/j.ijfoodmicro.2017.11.017, 29202339

[ref33] Tang H. Cao T. Liang X. Wang A. Salley S. O. McAllister J. . (2009). Influence of silicone surface roughness and hydrophobicity on adhesion and colonization of *Staphylococcus epidermidis*. J. Biomed. Mater. Res. A 88, 454–463. doi: 10.1002/jbm.a.31788, 18306290

[ref34] Tian X. Li T. Liu Y. Tian Y. Li Z. Wang Y. . (2025). Based on surface roughness and hydrophobicity to reduce the bacterial adhesion to collagen films for the ability to enhance the shelf life of food. Food Packag. Shelf Life 48:101473. doi: 10.1016/j.fpsl.2025.101473

[ref35] Tian P. Yang D. Mandrell R. (2011). Differences in the binding of human norovirus to and from romaine lettuce and raspberries by water and electrolyzed waters. J. Food Prot. 74, 1364–1369. doi: 10.4315/0362-028X.JFP-10-494, 21819668

[ref36] U.S. Centers for Disease Control and Prevention. (2024). Norovirus Facts and Stats. Atlanta, USA: CDC Norovirus. Available online at: https://www.cdc.gov/norovirus/data-research/index.html (Accessed August 22, 2025)

[ref37] Vega E. Garland J. Pillai S. D. (2008). Electrostatic forces control nonspecific virus attachment to lettuce. J. Food Prot. 71, 522–529. doi: 10.4315/0362-028X-71.3.522, 18389695

[ref38] Vega E. Smith J. Garland J. Matos A. Pillaii S. D. (2005). Variability of virus attachment patterns to butterhead lettuce. J. Food Prot. 68, 2112–2117. doi: 10.4315/0362-028x-68.10.2112, 16245715

[ref39] Velásquez P. Skurtys O. Enrione J. Osorio F. (2011). Evaluation of surface free energy of various fruit epicarps using acid–base and zisman approaches. Food Biophys. 6, 349–358. doi: 10.1007/s11483-011-9209-0

[ref40] Yi J. Leveau J. H. J. Nitin N. (2022). Role of multiscale leaf surface topography in antimicrobial efficacy of chlorine-based sanitizers. J. Food Eng. 332:111118. doi: 10.1016/j.jfoodeng.2022.111118

